# Biomolecular Condensates Integrate Transcriptional and Epigenetic Responses to Hypoxia

**DOI:** 10.3390/ijms27177926

**Published:** 2026-09-05

**Authors:** Chinmaya Kumar Patel, Ahmed Saif, Xiaojun Ren

**Affiliations:** Department of Biological Sciences, The University of North Carolina at Charlotte, Charlotte, NC 28223, USA

**Keywords:** hypoxia, HIF, IDR, phase separation, transcription, chromatin

## Abstract

Hypoxia is a defining feature of physiological stress and the core of solid tumors, where aberrant vascularization limits oxygen delivery; cells respond through mechanisms that extend beyond the canonical stabilization of hypoxia-inducible factors (HIFs). Recent studies suggest that hypoxia can promote the formation of specific biomolecular conden-sates, membraneless compartments generated through liquid–liquid phase separation in which regulatory proteins and RNAs become locally enriched at genomic regions, while chromatin mainly serves as an organizational scaffold. Transcription factors, the coacti-vators p300/CBP, Mediator, and BRD4, chromatin-modifying enzymes, and architectural RNAs such as NEAT1 and MALAT1 partition into these compartments, and their con-densation can help reorganize local chromatin structure and enhancer–promoter interac-tions. Because molecular oxygen is a shared co-substrate for the Jumonji-C histone demethylases and the ten-eleven translocation (TET) DNA dioxygenases, hypoxia reshapes histone methylation and DNA methylation in parallel, and readers that bridge these marks, including UHRF1, may participate in condensate-associated chromatin regulation. Hypoxia-driven condensation of ZHX2 rewires enhancer–promoter contacts and higher-order genome architecture, influencing cell identity, stemness, and metastatic potential, and Polycomb condensates represent another candidate epigenetic compartment that may be influenced by hypoxic signaling. These processes may be particularly important in cancer, where chronic hypoxia provides a sustained stimulus for condensate formation and epigenetic remodeling. Together, these findings support a model in which phase separation and epigenetic reprogramming are not separate layers but one integrated response to low oxygen, offering opportunities to target maladaptive condensates in disease.

## 1. Introduction

Hypoxia occurs in the human body when the supply of oxygen to tissues is insufficient to maintain adequate homeostasis [1,2]. This inadequate oxygen delivery to tissues results from either reduced blood flow or low blood oxygen content. Oxygen deprivation can severely affect various body cells that perform essential biological processes. Complete deprivation of oxygen supply in the body is called anoxia. Since oxygen is an important electron receptor for mitochondrial respiration and ATP production, the maintenance of oxygen homeostasis is a prerequisite for survival in eukaryotic cells [3]. The widespread contribution of hypoxia to disease pathogenesis has necessitated deep investigation into the cellular mechanisms of oxygen sensing. The cellular ability to sense and adapt to changes in oxygen availability is primarily orchestrated by a family of transcription factors known as Hypoxia-Inducible Factors (HIFs) [4]. While the anchoring of stabilized HIFs to genomic Hypoxia Response Elements (HREs) is critical for driving survival genes, this interaction acts only as a scaffold around which the broader machinery of gene regulation must assemble [5]. To successfully turn on genes, HIFs must manage a complicated process that involves three key steps: assembling the basic machinery to start transcription, opening up the tightly packed DNA structure, and controlling the speed of the enzyme (RNAPII) that actually reads the genetic code [6]. HIF turns on genes almost entirely by recruiting two specific helper proteins, CBP and p300, because these proteins help modify the DNA structure to allow reading [7]. It is well-established that HIFs employ a diverse array of transcriptional cofactors to circumvent rate-limiting steps in gene expression. This includes chromatin remodelers, such as the SWI/SNF complex and Pontin/Reptin, which modify nucleosome architecture to enhance DNA accessibility [8]. Consistent with a chromatin-based mechanism, the SWI/SNF (BAF) remodeling complex is specifically required for the HIF-1α-dependent hypoxic transcriptional response [8], and the related AAA+ ATPases Pontin and Reptin are reciprocally methylated under hypoxia to fine-tune the selection of HIF-1 target genes [9,10]. Additionally, the Mediator Kinase CDK8 is recruited to facilitate the release of paused RNA Polymerase II (RNAPII), ensuring productive elongation [11]. Furthermore, the identification of metabolic enzymes like PKM2 as non-canonical co-activators highlights an intrinsic coupling between cellular metabolism and the hypoxic transcriptional response [12]. Conversely, inhibition of the histone methyltransferase G9a alters PKM2 levels and autophagic signaling in glioma cells [13], indicating that the coupling between chromatin modifiers and glycolytic enzymes operates in both directions. More recent comprehensive reviews have synthesized the rapidly expanding understanding of oxygen sensing, HIF regulation and its therapeutic targeting, motivating the integration of these advances with emerging condensate-based mechanisms [14,15].

In 2019, Peter J. Ratcliffe, William G. Kaelin Jr., and Gregg L. Semenza were awarded the Nobel Prize in Physiology for their discovery of how cells regulate gene expression under different oxygen conditions by identifying hypoxia-inducible factor-1α (HIF-1α) and the mechanism through which it is selectively degraded [16]. HIFs govern the expression of hundreds of genes involved in erythropoiesis, angiogenesis, metabolic reprogramming, and cell survival. These proteins belong to the basic helix–loop–helix/PER-ARNT-SIM (bHLH-PAS) family and function as “master switches” for gene expression. Members of the human HIF family (HIF-1, HIF-2, and HIF-3) are heterodimeric transcription factors, each composed of an α and a β subunit [17,18]. The activity of this complex is tightly controlled by the stability of the HIF- α subunit. Under normal oxygen conditions (normoxia), the cell keeps HIF-α at very low levels through a rapid degradation system [19]. Oxygen allows specific enzymes to modify (hydroxylate) proline residues on the HIF-α protein [20]. This modification acts as a “destruction tag,” allowing the von Hippel-Lindau (VHL) tumor suppressor protein to recognize HIF-α and target it for proteasomal degradation. These oxygen-dependent prolyl-hydroxylation and VHL-capture steps were originally defined biochemically as the molecular basis of oxygen sensing [21,22,23]. A secondary checkpoint involves the factor inhibiting HIF (FIH-1), which blocks the machinery required for gene activation [24]. FIH-1 was first identified as an HIF-1α-interacting repressor [24] and was subsequently shown to be an asparaginyl hydroxylase that modifies Asn803 within the HIF-α C-terminal transactivation domain to prevent p300/CBP recruitment [25]. However, when oxygen levels drop (hypoxia), this destruction machinery is disabled. As a result, HIF-α stabilizes, accumulates in the nucleus, and pairs with the HIF-β subunit. This active complex then binds to specific DNA sequences to turn on genes critical for survival [26]. HIF-1α and HIF-2α are the primary activators of the hypoxic response, yet they regulate different sets of genes. Studies using “knockout” mice reveal that HIF-1α is essential for vascular development and embryonic survival [27]. In contrast, HIF-2α is more critical for regulating metabolism and organ function; its absence leads to multi-organ failure and metabolic disorders like hypoglycemia [28,29,30,31].

Dysregulation and overexpression of HIF, induced by genetic alterations and hypoxia, have been implicated in several pathophysiologies, including ischemia and cancer [32,33,34,35,36,37,38]. HIF accumulation has dual effects, including cell death and cell survival, in neurovascular diseases such as stroke, traumatic brain injury (TBI), and Alzheimer’s disease [2,39,40,41,42,43,44,45,46].

It has been believed that the cell’s oxygen response was just about a single protein (HIF) drifting around until it found the right gene to turn on, but this does not explain how the cell activates hundreds of genes simultaneously. It turns out that inside the cell, it undergoes liquid–liquid phase separation to form biomolecular condensates in response to hypoxia [47]. By gathering all the proteins into these liquid droplets, thereby increasing their local concentration, the cell can coordinate a massive response much faster than a single protein can.

Recent research has established that biological polymers, such as RNA and proteins, can spontaneously demix from the surrounding solution to form membraneless organelles. This phenomenon is known as liquid–liquid phase separation (LLPS). It results in the formation of functional condensates, such as the nucleolus and stress granules [48,49], which act as reaction crucibles by sequestering necessary components at specific intracellular locations, a principle that also extends to purified proteins—ranging from the intrinsically disordered α-synuclein to folded proteins such as serum albumin, transferrin, and trypsin—that undergo LLPS in vitro under macromolecular crowding [50,51,52,53,54]. Proteins prone to LLPS typically contain Intrinsically Disordered Regions (IDRs) rich in low-complexity sequences. These flexible domains drive condensate formation by enabling weak, nonspecific interactions that allow the protein to associate promiscuously with itself and other macromolecules [55,56,57,58]. In this review, we will discuss how studies on transcriptional and coactivator condensates, hypoxia-induced chromatin rewiring, epigenetic remodeling, phase-separated tumor suppressors, and RNA-scaffolded condensates collectively reveal that hypoxia-driven phase separation and epigenetic regulation orchestrate transcriptional, chromatin, and epitranscriptomic programs, ultimately controlling gene expression, cellular plasticity, cancer progression, and the tumor microenvironment. Rather than surveying phase separation in general, we specifically synthesize how oxygen sensing is mechanistically coupled to condensate formation across both the nuclear and cytoplasmic compartments—spanning transcriptional and coactivator hubs, Polycomb and other epigenetic condensates, RNA-scaffolded paraspeckles, and metabolic bodies—thereby framing hypoxia-regulated condensation, rather than any single factor such as ZHX2, as an integrated organizing principle of the hypoxic response.

## 2. Hypoxia-Induced Transcriptional Condensates

Many studies have highlighted that the main driver of the hypoxic response is the formation of super-enhancers by HIF proteins. This section discusses the biomolecular condensation mechanism.

Although the biophysical principles discussed below were largely established in general or stem-cell models, they are directly relevant to hypoxia because HIF activates transcription through precisely this condensate-forming machinery. Upon oxygen-dependent stabilization, HIF-α accumulates, dimerizes with HIF-1β, and binds hypoxia-response elements that are strongly enriched at active enhancers and super-enhancers genome-wide [59]. There, the HIF-α C-terminal transactivation domain docks onto the intrinsically disordered regions of the coactivators p300/CBP [7], which themselves co-condense with transcription factors to control the kinetics of transcriptional bursting [60]. HIF further recruits the CDK8-Mediator module to release paused RNA polymerase II and depends on SWI/SNF remodeling for productive target-gene activation [8]. Because p300/CBP, Mediator, BRD4, and RNA polymerase II are the very factors that partition into liquid-like transcriptional condensates at super-enhancers [61,62], hypoxia can be viewed as a physiological stimulus that nucleates and reorganizes these condensates at HIF target loci. The subsections below detail the IDR- and coactivator-driven condensation principles that this hypoxic program exploits.

### 2.1. The Role of IDRs in Transcription-Coupled Biomolecular Condensates

Transcription factors (TFs) are the molecular switches that control gene expression. They operate by scanning the genome and binding to specific, short DNA sequences known as motifs, typically 6–12 base pairs in length [63]. However, this creates a biological paradox: these short motifs appear thousands of times across the genome, yet a specific TF only binds to a tiny fraction of them. To understand how TFs select the right targets among a sea of potential binding sites, two main explanations have been proposed. The first is chromatin accessibility, the idea that TFs bind only where DNA is physically unpacked and exposed [64]. Although this is true, it fails to explain how closely related TFs that recognize the same DNA motif often regulate completely different sets of genes within the same accessible regions. The second explanation focuses on the DNA-Binding Domain (DBD), suggesting that the protein’s structured region might recognize flanking sequences or interact with specific cofactors to guide placement [65]. Traditionally viewed as unstructured tethering elements, intrinsically disordered regions (IDRs) in key transcriptional regulators such as BRD4 and the Mediator complex are now recognized as functional drivers of transcription [66,67]. This realization establishes a new paradigm linking the phase-separation properties of these low-complexity domains to the precise spatiotemporal control of the transcriptional machinery. A previous study shows that the low-complexity domains (LCDs) found within transcription factors are not inert; rather, they serve as sticky interfaces that organize proteins into concentrated hubs [68]. Through a series of dynamic, specific, multivalent interactions, these domains enable transcription factors to cluster. When a critical density is reached, these clusters can spontaneously transition into distinct liquid phases. Indeed, Sabari et al. showed that high BRD4 and Mediator density at super-enhancers drives liquid condensate formation [61]. By creating these concentrated, distinct compartments, the cell forms a localized hub of the transcriptional apparatus needed to sustain high-level expression of lineage-specifying genes. Further investigation by Cho et al. revealed that Mediator and RNA Polymerase II condensates are biochemically distinct, as evidenced by their differential responses to selective inhibitors. The authors propose a model in which dynamic exchange between these liquid phases facilitates the transition from initiation to productive elongation [62]. The study demonstrates that Mediator and Pol II co-localize within large, stable clusters that exhibit the liquid-like properties of biomolecular condensates, such as rapid component exchange and fusion, rather than functioning as static complexes. They proposed a dynamic kissing model where enhancer-bound Mediator condensates transiently contact gene promoters to initiate transcription, rather than maintain continuous contact. This phase separation framework explains how transcription factors can bridge large physical distances (>300 nm) between enhancers and promoters, potentially allowing a single Mediator condensate to regulate multiple genes simultaneously without requiring direct, permanent molecular adhesion. Further, Brodsky et al. investigated IDRs as the missing navigation system for transcription factors. They focused on Msn2 and Yap1, two yeast transcription factors that possess extensive disordered regions (over 500 amino acids long) [69]. In the hypoxic context, this IDR-based target-search logic is significant because the HIF-α subunits themselves carry extensive intrinsically disordered transactivation domains, implying that oxygen-regulated disordered interactions help direct HIF to the correct subset of genomic sites under low oxygen.

### 2.2. Coactivator Condensates and Gene Control

Super-enhancers (SEs) are critical regulatory elements that drive high-level transcription of genes essential for cell identity. These genomic regions are defined by an unusually high density of master transcription factors and coactivators, particularly BRD4 and MED1 [70,71]. Hnisz et al. have developed a simplified polymer physics model that represents enhancer components as interacting chains defined by three key parameters: concentration (N), valency (f), and the equilibrium interaction constant (Keq) [72]. Previously, the study also demonstrated that TGF-β, LIF, and Wnt signaling pathways are hubs that recruit exceptionally high densities of RNA Polymerase II, Cohesin, and chromatin remodelers, explaining their ability to produce high levels of enhancer RNA (eRNA) and robustly activate target genes [70].

Recent theoretical frameworks suggest that these high-density assemblies may not function as static complexes but rather as phase-separated biomolecular condensates [61]. Again, Xu et al. have studied p300 and CBP as functionally redundant coactivators due to the high structural conservation of their catalytic and protein-binding domains [73]. They explained that both proteins feature a central acetyltransferase (HAT) domain flanked by specific regulatory modules, including the Bromodomain, PHD, and TAZ2, which collectively govern substrate specificity and autoregulation. Further, they elaborated that while the catalytic cores of p300 and CBP are nearly identical, this structural conservation is starkly contrasted against the significant divergence of their intrinsically disordered regions (IDRs). They postulated that these non-conserved regions function as critical regulatory modules, modulating catalytic output through distinct mechanisms such as transcription factor-dependent biomolecular condensation. This is directly pertinent to hypoxia: p300 and CBP are the principal HIF coactivators, and their transcription-factor-dependent condensation [60] provides a mechanism by which stabilized HIF-α can locally concentrate acetyltransferase activity at hypoxia-response elements to drive rapid, switch-like induction of oxygen-regulated genes.

## 3. Hypoxia Rewires Chromatin Topology

### 3.1. Hypoxia-Driven Phase Separation: The ZHX2 Paradigm

**Figure 1:**
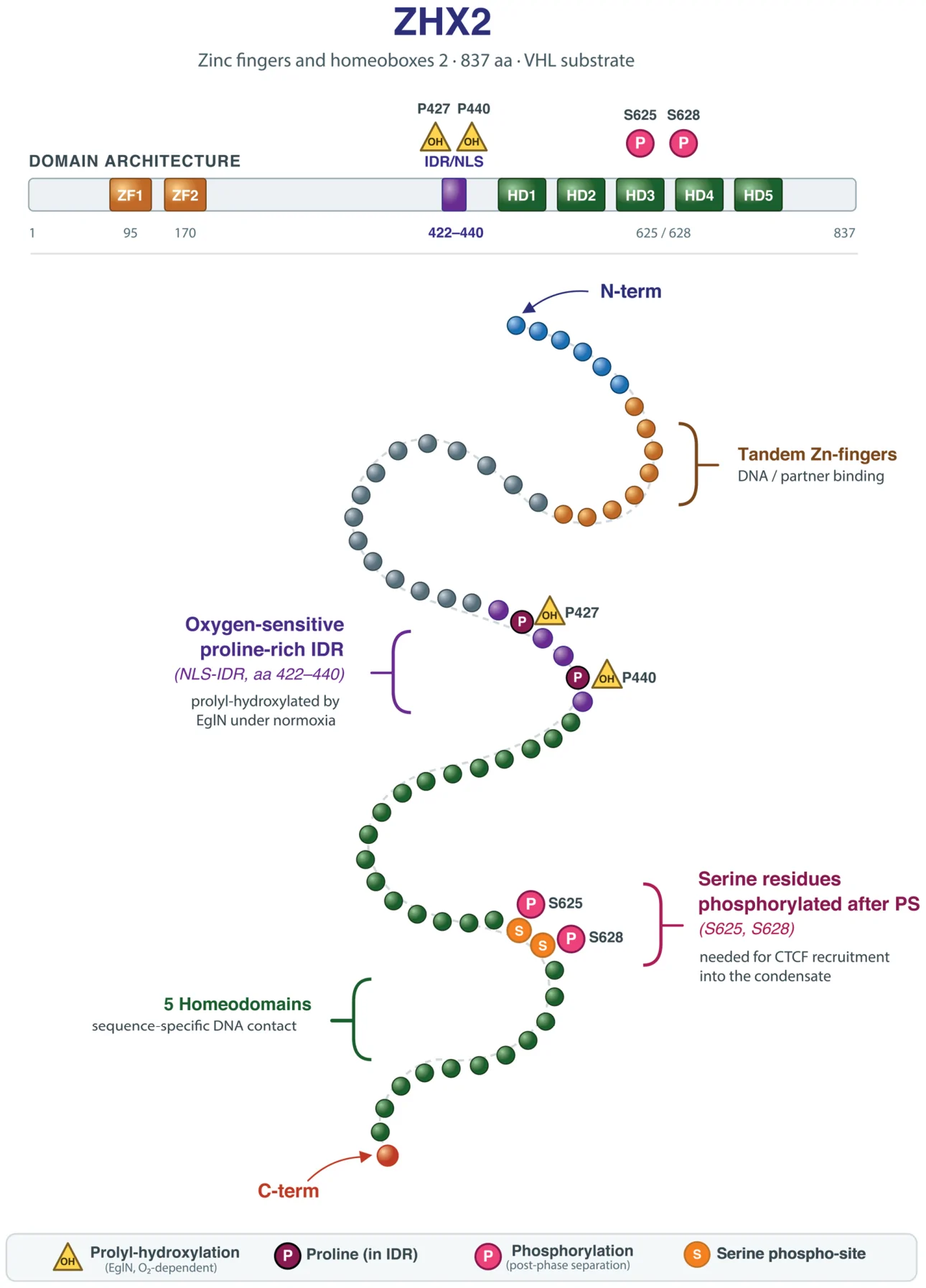
**A single intrinsically disordered region couples oxygen-responsive signaling and condensate formation in ZHX2.** Human ZHX2 (zinc fingers and homeoboxes 2) is a transcriptional regulator and a substrate of the von Hippel–Lindau (VHL) tumor suppressor. (**Top**) Schematic of the ZHX2 protein. Two tandem zinc fingers (ZF1, ZF2; approximately residues 95 and 170) and five homeodomains (HD1–HD5) provide DNA and partner binding and are separated by a proline-rich intrinsically disordered region that overlaps the nuclear localization signal (IDR/NLS, residues 422–440). Two sets of regulatory sites flank this region: the prolyl-hydroxylation acceptors Pro427 and Pro440 (yellow triangles, OH) and the phosphorylation sites Ser625 and Ser628 (magenta circles, P). (**Bottom**) The same protein is drawn as a folded polypeptide chain from the N- to the C-terminus, showing how the structured DNA-binding modules flank the disordered segment. The arrangement explains the protein’s regulatory logic. Under normoxic conditions, oxygen-dependent EglN prolyl hydroxylases hydroxylate Pro427 and Pro440, allowing this region of ZHX2 to function as an oxygen-responsive regulatory module. Ser625 and Ser628, by contrast, are phosphorylated only after phase separation has occurred and are required to recruit CTCF into the resulting condensate (Figure 2 and Figure 3). ZHX2 therefore contains both oxygen-responsive regulatory sites and the condensate-promoting IDR within the same short segment. *Abbreviations:* aa, amino acids; CTCF, CCCTC-binding factor; EglN, egg-laying-defective nine family prolyl hydroxylase; HD, homeodomain; IDR, intrinsically disordered region; NLS, nuclear localization signal; ZF, zinc finger.

Membrane-less organelles formed via LLPS have emerged as distinct compartments essential for regulating the flow of genetic information, influencing processes ranging from transcription and splicing to higher-order chromatin organization [74,75,76,77,78]. A salient example is Zinc Fingers and Homeoboxes 2 (ZHX2), a VHL substrate that directly links oxygen sensing to chromatin topology (Figure 1). More broadly, hypoxia rewires chromatin through two complementary routes: a HIF-dependent arm, in which stabilized HIF recruits chromatin modifiers and remodelers to target loci, and a HIF-independent arm, in which molecular oxygen itself is a rate-limiting substrate for chromatin-modifying dioxygenases (Section 3.2). ZHX2 illustrates how these routes converge on phase separation to reorganize genome topology.

**Figure 2:**
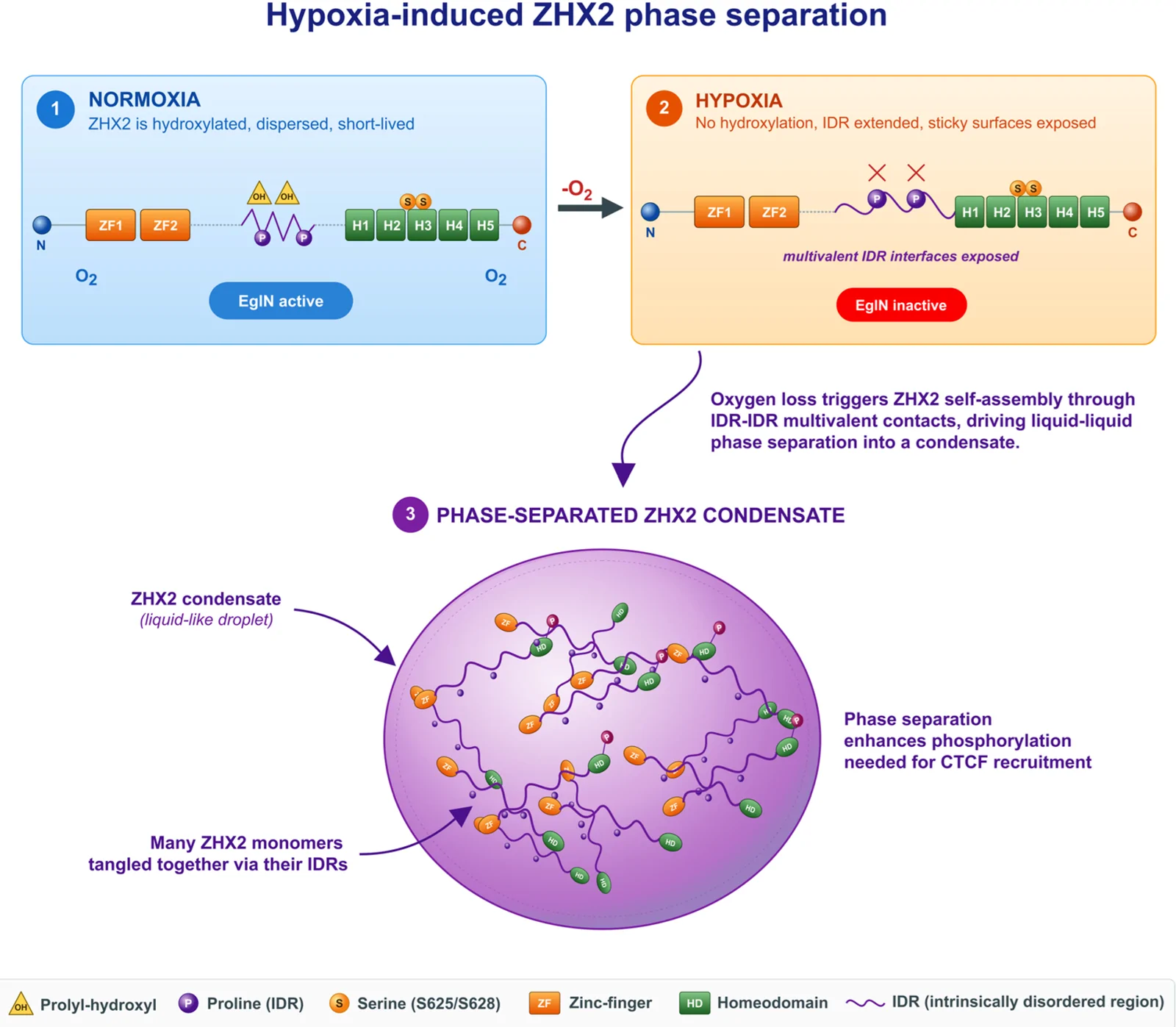
**Loss of prolyl-hydroxylation converts ZHX2 from a dispersed protein into a phase-separated condensate.** The three panels follow ZHX2 as oxygen levels decline. **(1)** Under normoxia, the EglN prolyl hydroxylases are active and modify Pro427 and Pro440 within the intrinsically disordered region (IDR) of ZHX2. The hydroxylated protein remains dispersed through the nucleoplasm and is short-lived, since this modification also permits its VHL-dependent turnover. The dashed lines indicate amino acid residues of ZHX2. **(2)** Under hypoxia, EglN activity fails for want of its oxygen co-substrate, and both hydroxylation events are lost (red crosses). The unmodified IDR relaxes into an extended conformation that exposes multiple weak, self-complementary interaction surfaces along its length. **(3)** These exposed surfaces allow many ZHX2 molecules to engage one another through multivalent IDR–IDR contacts, so that the protein demixes from the nucleoplasm into a liquid-like nuclear condensate in which it is locally concentrated. Phosphorylation of Ser625 and Ser628 is favored in this crowded environment and, in turn, permits CTCF recruitment (Figure 3). The scheme thus depicts an oxygen-gated switch in which a chemical modification at two proline residues determines whether ZHX2 remains soluble or assembles into a functional compartment. *Abbreviations:* CTCF, CCCTC-binding factor; EglN, egg-laying-defective nine family prolyl hydroxylase; H1–H5/HD, homeodomain; IDR, intrinsically disordered region; O_2_, molecular oxygen; ZF, zinc finger.

**Figure 3:**
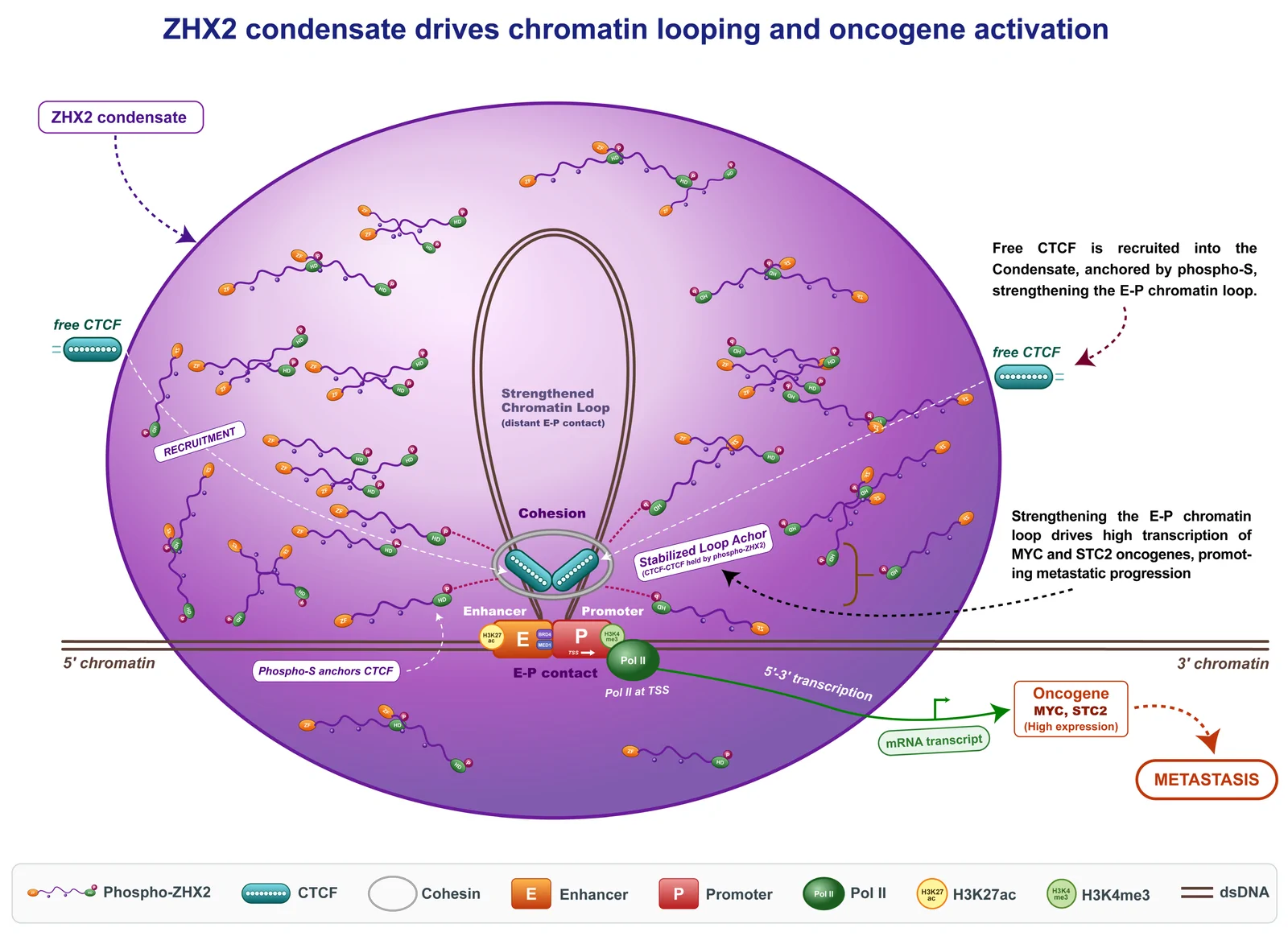
**Proposed model: the hypoxic ZHX2 condensate stabilizes an enhancer–promoter loop and drives oncogene transcription.** The condensate formed in Figure 2 (large purple sphere) concentrates phosphorylated ZHX2 and acts as a capture compartment for CTCF. Free CTCF molecules diffusing in the nucleoplasm partition into the condensate (white dashed arrows) and are retained there by the phospho-Ser625/Ser628 surfaces of ZHX2 (dark-red dashed arrows), raising the local CTCF concentration well above that of the surrounding nucleoplasm. Two such CTCF molecules pair at the base of a chromatin loop and, together with Cohesin, secure a contact between a distant enhancer and its promoter that would otherwise be transient. The enhancer (E) carries H3K27ac together with the coactivators BRD4 and MED1, the promoter (P) carries H3K4me3, and RNA polymerase II is engaged at the transcription start site, so stabilizing this contact sustains productive transcription (green arrow, 5′→3′) rather than merely permitting it. The functional consequence, indicated by the black dashed arrow, is high-level expression of the *MYC* and *STC2* oncogenes and, ultimately, metastatic progression (red dashed arrow). The model therefore connects the physical property of the condensate, its capacity to concentrate a scarce architectural protein, to a defined transcriptional and phenotypic outcome. *Abbreviations:* BRD4, bromodomain-containing protein 4; CTCF, CCCTC-binding factor; dsDNA, double-stranded DNA; E–P, enhancer–promoter; H3K27ac, histone H3 lysine 27 acetylation; H3K4me3, histone H3 lysine 4 trimethylation; MED1, Mediator complex subunit 1; Pol II, RNA polymerase II; STC2, stanniocalcin 2; TSS, transcription start site.

#### 3.1.1. Molecular Determinants of ZHX2 Condensation

Gao et al. have shown that ZHX2 undergoes a rapid phase transition into discrete nuclear puncta when oxygen levels drop below 2.5%, a threshold frequently observed in solid tumors [79]. This process is governed by a specific proline-rich intrinsically disordered region (IDR) located within the nuclear localization sequence (residues 422-440) (Figure 1). The mechanism relies on an “oxygen switch” embedded within the IDR. Under normoxia, prolyl hydroxylases (presumably EglNs) hydroxylate specific residues (P427 and P440), thereby maintaining ZHX2 in a dispersed state. Oxygen scarcity inhibits this hydroxylation, likely inducing a conformational shift that exposes multivalent interfaces and drives condensation (Figure 2). Mutational analyses support this: a “PA mutant” (proline-to-alanine conversion) abrogates condensation, underscoring the critical role of unmodified prolines in facilitating the weak, multivalent interactions required for phase separation.

#### 3.1.2. Rewiring Chromatin Topology for Metastasis

These condensates assemble on chromatin and serve as hubs for transcriptional hyperactivity. By concentrating transcriptional machinery (RNA Pol II-S5P, BRD4, MED1) and bringing distant genomic loci into proximity, ZHX2 condensates facilitate de novo chromatin loop formation [74,75,76,77,78,79]. Integration of Hi-C and ATAC-seq reveals that ZHX2-CTCF co-occupancy is enriched at enhancer–promoter (E-P) and enhancer-enhancer (E-E) loops. This reorganization is not random; it specifically targets oncogenic super-enhancers, driving the overexpression of metastatic drivers such as *MYC* and *STC2*.

These studies uncover a novel ZHX2-CTCF axis that functionally bridges environmental stress and epigenetic rewiring. Hypoxia mediates the phase separation of ZHX2 via its proline-rich intrinsically disordered region (IDR), creating a scaffold that recruits other transcriptional components [80]. This suggests a two-step molecular mechanism in which oxygen scarcity impairs prolyl-hydroxylation, driving condensation and subsequent phosphorylation, thereby facilitating CTCF partitioning into these condensates. By integrating imaging-based cellular experimentation with spatial transcriptomics and conformation capture technologies, they revealed how this hypoxia-induced condensate rewires global chromatin looping to activate a specific metastatic program (Figure 3).

### 3.2. Hypoxia-Driven Epigenetic Remodeling and Condensates

#### 3.2.1. Epigenetic Remodeling Under Hypoxia

A key mechanistic point is that oxygen can directly regulate the epigenome, independently of HIF. Jumonji-C (JmjC) histone demethylases are Fe (II)- and 2-oxoglutarate-dependent dioxygenases that use molecular oxygen as a co-substrate, and several members act as bona fide oxygen sensors. Hypoxia raises histone methylation, most notably H3K4me3, H3K9me3, H3K27me3, and H3K36me3, within minutes, before HIF target genes are fully induced, through direct inhibition of KDM catalytic activity [81]. In parallel, KDM6A/UTX and KDM5A were shown to sense oxygen directly, such that their reduced activity under hypoxia increases H3K27me3 and H3K4me3 at developmental loci and blocks cell differentiation even when HIF signaling is bypassed [81,82]. These oxygen-sensitive demethylases therefore complement HIF-dependent recruitment of chromatin modifiers, providing a rapid, HIF-independent arm of the hypoxic epigenetic response.

Epigenetic remodeling by hypoxia is the oxygen-dependent reprogramming of chromatin structure and gene expression through changes in DNA methylation, histone modifications, and chromatin organization. Hypoxia profoundly influences chromatin architecture and epigenetic regulation, thereby reshaping transcriptional programs that govern cell fate decisions [83]. A central mediator of epigenetic repression is the Polycomb Repressive Complex 2 (PRC2), a histone methyltransferase complex composed of SUZ12, EED, and the catalytic subunit EZH2 [84,85,86]. PRC2 dysregulation is frequently observed in cancer [87]. In many solid tumors, inactivating mutations in PRC2 subunits reduce global H3K27me3 levels, although focal redistribution of the mark often persists. Conversely, gain-of-function EZH2 mutations, particularly in lymphomas, increase H3K27me3 deposition and block differentiation. These observations suggest that oncogenic alterations in PRC2 do not merely change overall H3K27me3 abundance but instead redistribute repressive chromatin domains, thereby uncoupling differentiation control from growth regulation. Hypoxic conditions can increase genome-wide bivalent chromatin marking and alter PRC2 distribution, thereby influencing cellular reprogramming and lineage plasticity [88]. In muscle stem cells, hypoxia-associated epigenetic remodeling has been implicated in regulating differentiation trajectories and supporting regenerative capacity. Accumulating evidence indicates that hypoxia is a potent regulator of epigenetic landscapes and cellular reprogramming [89,90]. Beyond HIF-mediated transcriptional responses, low-oxygen conditions remodel chromatin through coordinated changes in histone acetylation, methylation, and DNA methylation, thereby influencing cell-fate plasticity. Watt et al. found that hypoxia reprograms chromatin to shift transcription start sites, remodeling 5′UTRs and selectively controlling protein translation independent of classical HIF1 pathways [91]. The study establishes a direct link between hypoxia-induced H3K4me3 redistribution and the selection of alternative TSSs, thereby remodeling transcription to support cancer cell survival and plasticity. Hypoxia has been shown to increase global H3K27me3 levels and expand the number of bivalent promoters in cancer cells, including MCF7 breast carcinoma cells [88]. Notably, hypoxia-induced enrichment of H3K4me3 within H3K27me3-occupied regions enhances bivalent chromatin marking, generating an epigenetic state reminiscent of that of embryonic stem cells [88,92]. Upon reoxygenation, many loci retain this bivalency, suggesting that hypoxia can imprint persistent epigenetic memory. Mechanistically, reduced activity of the H3K27me3 demethylase KDM6B/JMJD3 under hypoxia contributes to sustained H3K27me3 accumulation [93]. In parallel, hypoxia redistributes H3K4me3 across the genome, including at loci supporting proliferation and metabolic adaptation [81,91]. Chromatin changes coincide with increased expression of pluripotency-associated transcription factors such as Oct4, Sox2, and Nanog. These factors operate as a mutually reinforcing regulatory circuit together with Polycomb complexes and stem-cell microRNAs [94], and hypoxia has correspondingly been linked to enhanced self-renewal, suggesting functional convergence between oxygen sensing and stemness networks [89]. Under hypoxia, epithelial gene promoters (e.g., E-cadherin, plakoglobin) acquire increased H3K27me3 and reduced H3K4 acetylation, whereas mesenchymal gene promoters (e.g., N-cadherin, vimentin) lose H3K27me3 [89]. This coordinated redistribution of repressive and activating marks promotes mesenchymal identity. In glioma stem cells, hypoxia also enhances TET1 and TET3 activity, which in turn activate Oct4 and Nanog regulatory regions, further reinforcing stem-like phenotypes. Hypoxia additionally intersects with telomere biology during reprogramming. Telomerase reverse transcriptase (*TERT*), essential for pluripotency and induced pluripotent stem cell (iPSC) maintenance [95], is transactivated under hypoxia through HIF-1 binding to consensus motifs in the *TERT* promoter [96], linking oxygen sensing to telomere maintenance and long-term self-renewal capacity. These principles have concrete, cell-type-specific readouts: in muscle stem cells, hypoxia-associated chromatin remodeling biases differentiation trajectories and preserves regenerative capacity [89], whereas in tumor-associated macrophages, hypoxia engages an NF-κB- and TET2-dependent program that demethylates and activates inflammatory and immunosuppressive gene networks, overriding microenvironmental cues [90]. Thus, hypoxia reshapes the epigenome not through a single mark but through coordinated rewiring of writers, erasers, and readers.

#### 3.2.2. Epigenetic Condensates Formed During Hypoxia

Beyond its spatial arrangement into higher-order chromatin domains, emerging evidence indicates that chromatin is further compartmentalized within membraneless nuclear structures formed through liquid-like behavior and phase separation mechanisms (Figure 2) [97,98,99,100]. Studies have demonstrated their involvement in the epigenetic regulation of gene expression in cancer (Figure 3). Shi et al. have demonstrated that UTX exerts its tumor-suppressive function through phase separation-mediated chromatin regulation [101]. A central intrinsically disordered region (cIDR) of UTX drives the formation of liquid-like condensates. Notably, the most common cancer-associated mutations disrupt this cIDR, impair condensate formation, and consequently abolish tumor-suppressive activity. Crucially, UTX/KDM6A is itself an oxygen-sensitive demethylase [82]; because hypoxia both suppresses its catalytic activity and can modulate the disordered cIDR that drives its condensation, we propose that low oxygen may reversibly tune UTX condensate formation and its tumor-suppressive output, thereby tying this phase-separation switch to the hypoxic tumor microenvironment. We emphasize that this connection was inferred from two independent lines of evidence and has not yet been demonstrated directly. Xiao et al. have highlighted key features and experimental approaches used to study phase separation and discuss how LLPS-mediated chromatin and transcriptional regulation may control CSC stemness [102]. Because hypoxia is a defining feature of the cancer–stem-cell niche and HIF signaling is a principal driver of stemness, the phase-separation events proposed to govern CSC identity are expected to operate within, and be reinforced by, this low-oxygen environment. Zhang et al. have shown that TiPARP functions as a transcriptional repressor of major regulatory factors such as HIF-1, c-Myc, and estrogen receptor. They showed that TiPARP undergoes ADP-ribosylation-dependent phase separation to form nuclear condensates, highlighting a regulatory link between post-translational modification and condensate assembly [103]. This example is explicitly hypoxia-linked: TiPARP/PARP7 is itself a HIF-1 target gene, so its condensate-dependent, ADP-ribosylation-driven degradation of HIF-1α establishes an oxygen-responsive negative-feedback loop that restrains the hypoxic transcriptional program.

A particularly direct bridge between hypoxic epigenetic remodeling and phase separation is provided by the Polycomb system. The PRC1 subunit CBX2 assembles nuclear condensates through a charged intrinsically disordered region, and this phase separation concentrates DNA and nucleosomes to promote chromatin compaction and gene silencing [104,105,106]. Because hypoxia increases global H3K27me3 and expands Polycomb-marked bivalent domains (Section 3.2.1), the low-oxygen state is expected to favor the assembly and stabilization of Polycomb condensates, providing a mechanistic route by which oxygen availability tunes repressive chromatin compartments. This example underscores that hypoxia-responsive epigenetic regulation and biomolecular condensation are two facets of one organizing principle that extends well beyond the ZHX2 paradigm.

#### 3.2.3. Crosstalk Between DNA Methylation and Histone Modifications in Hypoxic Condensates

The preceding sections considered histone modifications and chromatin architecture, but hypoxia also reshapes DNA methylation, and the two systems are mechanistically coupled. The ten-eleven translocation (TET) enzymes that initiate DNA demethylation by oxidizing 5-methylcytosine (5mC) are, like the JmjC histone demethylases, Fe (II)- and 2-oxoglutarate-dependent dioxygenases that consume molecular oxygen as a co-substrate. Thienpont et al. showed that tumor hypoxia directly reduces TET catalytic activity, and that this occurs independently of hypoxia-associated changes in TET expression, proliferation, metabolism, HIF activity, or reactive oxygen species [107]. The consequence is increased promoter hypermethylation: in patient tumors, tumor-suppressor promoters are markedly more methylated in hypoxic regions, and the authors estimated that up to half of all hypermethylation events may be attributable to oxygen shortage. Hypoxia therefore acts on the epigenome through a single unifying principle: oxygen is rate-limiting for an entire class of chromatin-modifying dioxygenases, so repressive histone methylation and DNA methylation increase in parallel rather than independently.

Condensation provides a physical mechanism by which these two marks are read together. UHRF1 is the archetypal example: its tandem Tudor domain binds H3K9me3, its SET- and RING-associated (SRA) domain recognizes hemi-methylated CpG, and it recruits DNA methyltransferase 1 (DNMT1) to propagate methylation after replication [108]. Recent work shows that UHRF1 also undergoes phase separation, driven by its SRA motif and a third intrinsically disordered region, forming nuclear condensates at the promoters of cancer-related genes into which DNMT1 is recruited; dissolving these condensates with 1,6-hexanediol weakens the UHRF1-DNMT1 interaction, reduces DNMT1 occupancy at CpG islands, and lowers promoter methylation [109]. Within such a compartment, a histone mark and a DNA mark are not merely correlated but are physically co-concentrated together with the enzyme that writes one of them, making UHRF1 condensates a direct molecular bridge between histone and DNA methylation. A comparable logic has been proposed for MeCP2, which reads 5mC and was reported to partition into heterochromatin condensates that are disrupted by Rett-syndrome mutations [110]. That interpretation, however, is contested by evidence that MeCP2 binds methylated DNA and forms nuclear foci independently of phase separation and heterochromatin organization [111]. We highlight this disagreement deliberately because it illustrates a caution that applies throughout this field: co-localization within a nuclear focus is not, by itself, evidence of liquid–liquid phase separation. Another example of DNA-histone coupling is N6-methyladenine (6mA), a non-canonical DNA modification that is markedly elevated in glioblastoma, where it co-localizes with H3K9me3-marked heterochromatin and is erased by the dioxygenase ALKBH1; ALKBH1 depletion reduces chromatin accessibility and silences oncogenic transcriptional programs [112]. Because ALKBH1, like the TET and JmjC enzymes, is a Fe (II)- and 2-oxoglutarate-dependent dioxygenase, 6mA is in principle also oxygen-sensitive, although direct hypoxic regulation of this mark has not yet been demonstrated.

In a hypoxic context, these observations predict that low oxygen should favor the assembly of methylation-reading condensates because hypoxia simultaneously elevates H3K9me3 and 5mC, the two ligands UHRF1 engages through its Tudor and SRA domains. The relationship is nonetheless context-dependent: in glioma stem cells, hypoxia has instead been reported to enhance TET1 and TET3 activity at *OCT4* and *NANOG* regulatory regions (Section 3.2.1), indicating that the net direction of DNA methylation change under hypoxia depends on cell type, on the severity and duration of oxygen deprivation, and on the availability of 2-oxoglutarate and related metabolites. Defining when hypoxia produces net hypermethylation versus locus-specific demethylation, and how condensation shifts that balance, remains an important open question and is now experimentally possible using condensate-disrupting and 2-oxoglutarate-modulating approaches.

## 4. RNA-Scaffolded Condensates: The Role of *NEAT1*

Dynamic RNA N6-methyladenosine (m6A) modifications enable cancer cells to rapidly adapt to microenvironmental changes [113]. In glioblastoma multiforme (GBM), hypoxia induces the m6A demethylase ALKBH5, which correlates with a hypoxia-associated gene signature in patient tumors [114]. Loss or inactivation of ALKBH5 suppresses hypoxia-driven recruitment of tumor-associated macrophages (TAMs) and immunosuppression, in part through reduced expression and secretion of *CXCL8*/IL8. ALKBH5 does not directly demethylate *CXCL8* mRNA; instead, it removes m6A marks from the lncRNA *NEAT1*, stabilizing *NEAT1* and promoting paraspeckle formation. This process sequesters the transcriptional repressor SFPQ away from the *CXCL8* promoter, enhancing *CXCL8* transcription. Re-expression of *CXCL8* in ALKBH5-deficient cells partially restores TAM recruitment and tumor progression. Overall, this study links hypoxia-driven epitranscriptomic remodeling to the establishment of an immunosuppressive tumor microenvironment in GBM. Studies show that noncoding RNAs, including lncRNAs, miRNAs, circRNAs, piRNAs, and others, can function as scaffolds, regulators, or cargo within liquid–liquid phase-separated (LLPS) condensates, modulating both physiological cellular organization and pathological processes [115].

*NEAT1* is itself a central node linking hypoxia to condensate biology. It is a direct transcriptional target of HIF, regulated principally by HIF-2, and hypoxic induction of *NEAT1* is sufficient to drive de novo paraspeckle formation and to promote cancer-cell survival [116]. Mechanistically, the long *NEAT1_2* isoform acts as an architectural RNA whose modular domains nucleate paraspeckles through liquid–liquid phase separation, recruiting the RNA-binding proteins NONO and SFPQ via their disordered and dimerization domains into a shell-and-core condensate [117]. Because m6A controls *NEAT1* stability, the hypoxia-induced eraser ALKBH5 increases *NEAT1* levels and paraspeckle number, directly coupling the oxygen-sensitive epitranscriptome to the assembly of a specific membraneless organelle [114,117].

Beyond *NEAT1*, additional nuclear lncRNAs connect hypoxia to condensate-based gene control. The speckle-associated lncRNA *MALAT1* is induced by hypoxia [118] and promotes the condensation of the splicing factor SRSF1 near nuclear speckles; these *MALAT1*-SRSF1 condensates are preferentially engaged by elongating RNA polymerase II and reprogram the alternative splicing of hypoxia-responsive, speckle-proximal genes, providing a direct mechanistic link between a hypoxia-induced lncRNA, phase separation and cancer-relevant RNA processing [119]. Together with *NEAT1*, these examples establish RNA scaffolds as tunable organizers of hypoxic condensates.

The epitranscriptome adds a further, hypoxia-sensitive layer of condensate regulation. N6-methyladenosine marks act as multivalent docking sites for the reader proteins YTHDF1-3, and m6A-rich transcripts markedly enhance the liquid–liquid phase separation of these readers, partitioning modified mRNAs into P-bodies, stress granules, and neuronal RNA granules [120]. Consistently, m6A-binding YTHDF proteins promote stress-granule assembly by lowering the saturation concentration for phase separation of core stress-granule proteins [121]. Because hypoxia reshapes the m6A landscape, chiefly through induction of the demethylase ALKBH5 [114], oxygen availability can remodel the composition and dynamics of RNA condensates, positioning m6A as a key intermediary between the hypoxic signal and the physical organization of the transcriptome.

Beyond long noncoding RNAs, other noncoding RNA classes participate in hypoxic condensate biology. Among microRNAs, miR-210 is the prototypical hypoxamiR: HIF-1 binds a hypoxia-response element in the miR-210 promoter to induce it across tumor types [122], and miR-210 restrains mitochondrial respiration, modulates DNA repair, and promotes angiogenesis [123]. The silencing machinery through which such microRNAs act is itself compartmentalized, because Argonaute and TNRC6/GW182 proteins concentrate in processing bodies, condensates in which translationally repressed messenger RNAs accumulate [124]. Phase separation also determines microRNA fate: YBX1 condensates selectively recruit miR-223 and sort it into exosomes, and point mutations that block YBX1 condensation impair this sorting [125]. Because hypoxia remodels stress granules and processing bodies (Section 5), oxygen availability likely governs where, and how efficiently, microRNA-mediated silencing occurs, although direct measurements of miRISC partitioning in hypoxic cells remain scarce.

Circular RNAs (circRNAs) provide a second and complementary link. Many circRNAs are hypoxia-responsive: *circDENND4C*, for example, is induced in breast cancer cells in a HIF-1alpha-dependent manner and supports proliferation, and its depletion suppresses glycolysis, migration, and invasion under low oxygen through up-regulation of miR-200b/c [126,127]. Independently of the hypoxia literature, circRNAs have emerged as efficient condensate scaffolds, because their covalently closed structure resists exonucleolytic decay [128] and present multivalent protein-binding surfaces [115,128]. The nuclear circRNA *circASH2* promotes liquid–liquid phase separation of Y-box binding protein 1 (YBX1), accelerating decay of tropomyosin 4 transcripts and thereby remodeling the cytoskeleton and suppressing liver cancer metastasis [129], whereas *circVAMP3* acts as a molecular scaffold that drives CAPRIN1 condensation and stress-granule assembly, suppressing c-Myc translation [130]. The convergence of these two studies, hypoxia-induced circRNAs on the one hand and circRNA-scaffolded condensates on the other, identifies circRNAs as strong candidate organizers of the hypoxic condensate landscape; establishing directly whether specific hypoxia-induced circRNAs nucleate condensates in low oxygen is a clear experimental priority.

## 5. Cytoplasmic and Metabolic Condensates in the Hypoxic Response

The condensates discussed so far are predominantly nuclear, yet hypoxia also reorganizes the cytoplasm through phase separation. Under low oxygen, glycolytic enzymes coalesce into non-membranous ‘G bodies’ in both yeast and human hepatocarcinoma cells, and cells unable to form these condensates divide abnormally, directly coupling condensation to the metabolic rewiring that sustains cells in hypoxia [131]. Hypoxia and the accompanying translational arrest likewise promote assembly of cytoplasmic stress granules; in tumors, these G3BP1-nucleated condensates sequester untranslated mRNAs and, upon reoxygenation, disassemble to release HIF-1-regulated transcripts, linking stress-granule dynamics to the oxygen-sensitive translational program [132].

Cytoplasmic condensation also intersects with the epitranscriptome: during hypoxia, the lncRNA *KB-1980E6.3* recruits the m6A reader IGF2BP1 into condensates that stabilize c-Myc mRNA and sustain breast cancer stem cells, mirroring the m6A and RNA-scaffolded mechanisms described in Section 4 [133]. Taken together, these nuclear, cytoplasmic, and metabolic examples suggest that hypoxia does not act through a single condensate or factor but deploys phase separation as a general strategy to co-regulate transcription, chromatin, RNA processing, and metabolism in a coordinated adaptive response.

## 6. Conclusions

Hypoxia orchestrates a multifaceted cellular response that extends far beyond the classical stabilization of HIF transcription factors. Emerging evidence shows that hypoxia drives biomolecular condensate formation through LLPS, coordinating transcriptional activation, chromatin remodeling, and epitranscriptomic regulation. IDRs in transcription factors, coactivators, and epigenetic regulators facilitate dynamic condensate assembly, concentrating the molecular machinery needed for rapid, precise gene expression. Concurrently, hypoxia-induced chromatin rewiring, mediated by factors such as ZHX2 and PRC2, reshapes higher-order genomic architecture, establishing permissive or repressive states that influence cell identity, stemness, and metastatic potential. RNA scaffolds, exemplified by *NEAT1*, further refine this spatial and functional organization, linking epitranscriptomic modifications to transcriptional control and tumor microenvironment remodeling. Together, these findings support a model in which hypoxia-induced phase separation and epigenetic remodeling function as interconnected regulatory platforms. Through their coordinated action, they reorganize gene expression networks that govern cellular plasticity, tumor progression, and adaptation to environmental stress. Elucidating these mechanisms provides a conceptual and translational framework for developing therapeutic strategies that selectively target aberrant condensates and dysregulated epigenetic programs in disease.

## Data Availability

No new data were created or analyzed in this study. Data sharing is not applicable to this article.
